# The prevalence of sleep disturbances among physicians and nurses facing the COVID-19 patients: a systematic review and meta-analysis

**DOI:** 10.1186/s12992-020-00620-0

**Published:** 2020-09-29

**Authors:** Nader Salari, Habibolah Khazaie, Amin Hosseinian-Far, Hooman Ghasemi, Masoud Mohammadi, Shamarina Shohaimi, Alireza Daneshkhah, Behnam Khaledi-Paveh, Melika Hosseinian-Far

**Affiliations:** 1 Department of Biostatistics, School of Health, Kermanshah University of Medical Sciences, Kermanshah, Iran; 2 Sleep Disorders Research Center, Kermanshah University of Medical Sciences, Kermanshah, Iran; 3 Department of Business Systems & Operations, University of Northampton, Northampton, UK; 4 Department of Nursing, School of Nursing and Midwifery, Kermanshah University of Medical Sciences, Kermanshah, Iran; 5 Department of Biology, Faculty of Science, University Putra Malaysia, Serdang, Selangor Malaysia; 6 School of Computing, Electronics and Maths, Coventry University, London, UK; 7 Department of Food Science & Technology, Ferdowsi University of Mashhad (FUM), Mashhad, Iran

**Keywords:** Sleep disturbances, COVID-19, Coronavirus, Nurses, Physicians, Healthcare workers

## Abstract

**Background:**

In all epidemics, healthcare staff are at the centre of risks and damages caused by pathogens. Today, nurses and physicians are faced with unprecedented work pressures in the face of the COVID-19 pandemic, resulting in several psychological disorders such as stress, anxiety and sleep disturbances. The aim of this study is to investigate the prevalence of sleep disturbances in hospital nurses and physicians facing the COVID-19 patients.

**Method:**

A systematic review and metanalysis was conducted in accordance with the PRISMA criteria. The PubMed, Scopus, Science direct, Web of science, CINHAL, Medline, and Google Scholar databases were searched with no lower time-limt and until 24 June 2020. The heterogeneity of the studies was measured using I^2^ test and the publication bias was assessed by the Egger’s test at the significance level of 0.05.

**Results:**

The I^2^ test was used to evaluate the heterogeneity of the selected studies, based on the results of I^2^ test, the prevalence of sleep disturbances in nurses and physicians is I^2^: 97.4% and I^2^: 97.3% respectively. After following the systematic review processes, 7 cross-sectional studies were selected for meta-analysis. Six studies with the sample size of 3745 nurses were examined in and the prevalence of sleep disturbances was approximated to be 34.8% (95% CI: 24.8-46.4%). The prevalence of sleep disturbances in physicians was also measured in 5 studies with the sample size of 2123 physicians. According to the results, the prevalence of sleep disturbances in physicians caring for the COVID-19 patients was reported to be 41.6% (95% CI: 27.7-57%).

**Conclusion:**

Healthcare workers, as the front line of the fight against COVID-19, are more vulnerable to the harmful effects of this disease than other groups in society. Increasing workplace stress increases sleep disturbances in the medical staff, especially nurses and physicians. In other words, increased stress due to the exposure to COVID-19 increases the prevalence of sleep disturbances in nurses and physicians. Therefore, it is important for health policymakers to provide solutions and interventions to reduce the workplace stress and pressures on medical staff.

## Background

In December 2019, severe and unknown cases of pneumonia were reported in Wuhan, China. The new disease was caused by a new type of coronavirus. The Virus spread rapidly throughout China and other parts of the world. On 30th January 2020, due to the growing number of infections in China and other parts of the world, the World Health Organization’s Emergency Committee announced the state of emergency [1–3]. The Virus - now known as SARS-CoV-2 – and its outbreak prompted the World Health Organization to categorize the crisis as a pandemic on 11th March 2020. The rapid spread of the Virus, the lack of definitive treatment, and the severity of the disease in some of the patients during the clinical treatment are now resulting in thousands of deaths every day. These have urged many countries to prepare for the worst, resulting in numerous instances of local and national lockdowns and also consequential economic burdens [4–7]. SARS-CoV-2 is a member of the Coronaviridae family; The RNA length in these single-stranded viruses is 26-32 (kb), and they are categorized into four genera of alpha (α), beta (β), gamma (γ) and delta (δ). HCoV-229E, HCoV-NL63, HCoV-OC43, HCoV-HKU1 cause infection and colds in immunosuppressed individuals. On the other hand, other viruses in this family, which include SARS-CoV, SARS-CoV-2, and MERS-CoV, cause other human infections with variable clinical severity, including respiratory disorders [8–10].

The SARS-CoV-2 virus primarily affects the human respiratory system. Fever, cough, fatigue, and myalgia are the most common symptoms. Other symptoms in these patients include headache, bleeding, and diarrhea. Decreased white blood cell counts and lymphopenia are also seen in most inpatients in intensive care. In general, the COVID-19 infection leads to a dangerous disease that affects various systems in human body, such as the cardiovascular, respiratory, nerve, blood circulation, and immune systems [6, 11]. Apart from the physical impacts, the disease has also impacted the mental health of patients, and people suspected of having the disease, and has caused psychological disorders such as loneliness, sleep disturbances, fear, anxiety, and depression [12].

If complications arise, the COVID-19 patients end up in a critical condition and require intensive care. Treating these patients expose the medical staff to a challenging task that requires repeated challenging actions and a high level of care. The stress caused in such cases increase the risk of physical and mental disorders in the healthcare staff [13]. Stress, anxiety, and depression in the face of a crisis are partly considered normal emotional reactions. However, health care workers in the past epidemics such as influenza H1N1 and severe acute respiratory syndrome (SARS) have experienced high levels of stress, anxiety, and depression, and have also shown the post-traumatic stress disorder (PTSD) symptoms [14].

Existing literature have reported that the prevalence of psychological symptoms in medical health workers during the COVID-19 epidemic is higher than in the past epidemics [15]. Increased concerns in the medical staff and their families can have a negative impact on the provision of health services, which can subsequently discourage and isolate the patients [16]. Statistics provided in China show that health workers in Wuhan, as the initial epicentre of COVID-19, experienced high levels of anxiety, depression, fear, anger, and stress, due to excessive work pressure, direct exposure to disease, and the possibility of infection [17, 18]. Stress is known to be a major cause of sleep disturbances in medical staff [16]. A study by Huang & Zhao (2020) reported a 23.6% increase in the prevalence of sleep disturbances in the COVID-19 medical staff, and this was higher than the prevalence of sleep disturbances in other community groups [19].

**Fig. 1:**
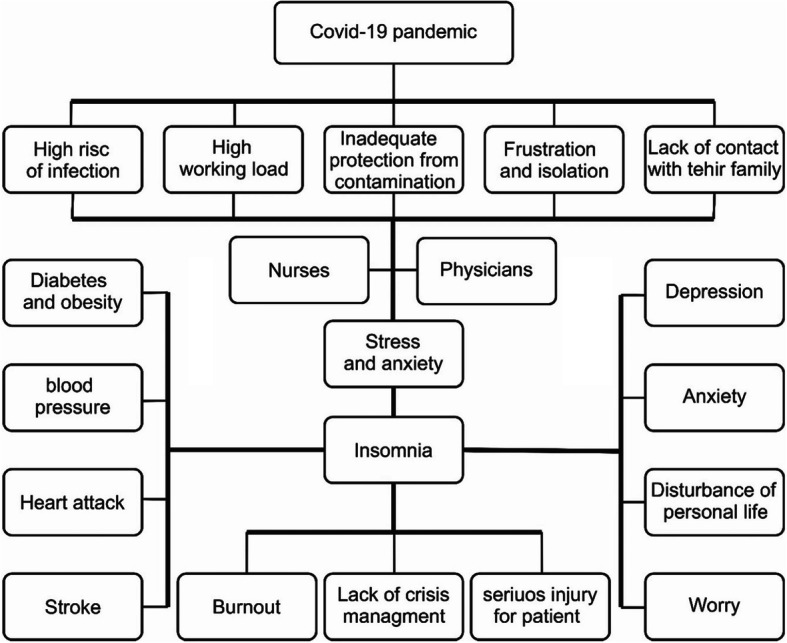
Impacts of the COVID-19 pandemic on sleep disturbances among medical staff

Today, sleep disturbances is known to be one of the most important concerns in public health. This disorder has a negative impact on the quality of life of millions of people globally [20]. Lack of sleep has significant negative effects on personal life and results in a reduction of physical activities [21]. Sleep disturbances is associated with a variety of physical complications, including increased risk of obesity, diabetes, high blood pressure, increased heart rate, heart attack, and stroke [22]. On the other hand, good quality sleep can swiftly improve the body’s function, relieve work-related fatigue, preserve energy levels, and maintain psychological health [18]. Due to the nature of the nurses and medical staff working conditions e.g. consecutive shifts, sleep disturbances have high prevalence among healthcare staff, and causes several psychological disorders in the day-to-day activities of these groups [23]. Patients with sleep disturbances suffer from more anxiety and depression and are less efficient. This reduction in occupational productivity among medical staff has irreversible consequences, with severe instances such as errors due to fatigue leading to a patient’s death. On the other hand, these conditions increase job burnout and severely affect the efficiency of health professionals in critical situations [21] (Fig. 1).

Given that maintaining the health of medical staff in crises such as the COVID-9 pandemic is crucial, and since determining the prevalence of physical and mental disorders in health workers for planning suitable interventions is very important, this study aims to examine the prevalence of sleep disturbances in nurses and physicians during the COVID-19 pandemic.

## Methods

### Protocol and information sources

This systematic review and meta-analysis were conducted according to the PRISMA (Preferred Reporting Items for Systematic review and Meta-Analysis) guidelines, with a view to examine the prevalence of sleep disturbances in nurses and physicians during the COVID-19 pandemic. As part of the research method, the databases of PubMed, Scopus, Science Direct, Web of science, CINHAL (with EBSCOHOST), and Medline (with EBSCOHOST) were searched with no lower time-limit and until 26 June 2020.

### Eligibility criteria

The inclusion and exclusion criteria were selected based on the PICO guidelines, according to this method, all physicians and nurses working in hospitals were the study population in this study. The intervention included physicians and nurses who were associated with patients with COVID-19 and the variable considered in this study, sleep disturbances were present in these physicians and nurses. in order to find the most relevant studies. Studies were deemed eligible that had considered physicians and nurses as the participants. Moreover, interventional studies were no eligible for the systematic review and meta-analysis. The comparisons were made on the studies that examined sleep disturbances in physicians and nurses. Research works with an outcome on the prevalence of sleep disturbances in nurses and physicians in the face of COVID-19 were considered as eligible.

The criteria for including studies were: cross-sectional studies, articles with their full-text available Moreover, the exclusion criteria include case control and cohort studies, case series, case reports, review papers and studies with unavailable full-text***.***

### Search strategy

In this study, search using keywords of health personnel, “healthcare workers”, “medical staff”, “healthcare professionals”, “emergency medical technicians”, nurse, physicians, covid-19, “covid-19 pandemic”, “sars-cov-2 infection”, “ coronavirus disease ”,“ sleep wake disorder ”,“ sleep disorder ”, sleep,“ Sleep Initiation and Maintenance Disorders ”, sleep disturbances, DIMS,“ sleep quality ”,“ sleep hygiene ” Done. Table 1 describes the strategies used in the various databases.

**Table 1:** Search Strategies

Data base	Search type	Search strategy	Date	number
PubMed	Basic search	in physicians: (health personnel[title/abstract] or “healthcare professionals”[title/abstract] or physicians[title/abstract]) and (covid-19[title/abstract] or “covid-19 pandemic”[title/abstract] or sars-cov-2 infection[title/abstract] or “coronavirus disease”[title/abstract]) and (sleep wake disorder[mesh] OR sleep disorder[title/abstract] or sleep[title/abstract] OR “Sleep Initiation and Maintenance Disorders”[mesh] OR insomnia[title/abstract] OR DIMS[title/abstract])	26.06.2020	31
		in Nurses: (health personnel[title/abstract]t or “healthcare workers”[title/abstract] or “medical staff”[title/abstract] or “emergency medical technicians”[title/abstract] or nurse[title/abstract] and (covid-19[title/abstract] or “covid-19 pandemic”[title/abstract] or sars-cov-2 infection[title/abstract] or “coronavirus disease”[title/abstract]) and (sleep wake disorder[mesh] OR sleep disorder[title/abstract] or sleep[title/abstract] OR “Sleep Initiation and Maintenance Disorders”[mesh] OR insomnia[title/abstract] OR DIMS[title/abstract])		
Web of Science	Advanced search	In physicians: title/abstract = (“medical staff” or “emergency medical technicians” or physicians) and title/abstract = (covid-19 or “covid-19 pandemic” or “sars-cov-2 infection” or “coronavirus disease”) and title/abstract = (“sleep wake disorder” OR “sleep disorder” or sleep OR “Sleep Initiation and Maintenance Disorders” OR insomnia OR DIMS OR “sleep quality” OR “sleep hygiene”)	26.06.2020	12
		In nurses: title/abstract = (health personnel or “healthcare workers” or “healthcare professionals” or “emergency medical technicians” or nurse) and title/abstract = (covid-19 or “covid-19 pandemic” or “sars-cov-2 infection” or “coronavirus disease”) and title/abstract = (“sleep wake disorder” OR “sleep disorder” or sleep OR “Sleep Initiation and Maintenance Disorders” OR insomnia OR DIMS OR “sleep quality” OR “sleep hygiene”)		
Scopus	Advanced search	In physicians: title/abstract -Keyword (health personal or “healthcare professionals” or “emergency medical technicians” or physicians) and title/abstract -Keyword (covid-19 or “covid-19 pandemic” or “sars-cov-2 infection” or “coronavirus disease”) and title/abstract -Keyword (“sleep wake disorder” OR “sleep disorder” or sleep OR “Sleep Initiation and Maintenance Disorders” OR “sleep quality” OR “sleep hygiene”)	26.06.2020	41
		In nurses: title/abstract -Keyword (“healthcare workers” or “medical staff” or “healthcare professionals” or “emergency medical technicians” or nurse) and title/abstract -Keyword (covid-19 or “covid-19 pandemic” or “sars-cov-2 infection” or “coronavirus disease”) and title/abstract -Keyword (“sleep wake disorder” OR “sleep disorder” or sleep OR “Sleep Initiation and Maintenance Disorders” OR “sleep quality” OR “sleep hygiene”)		
Pro Quest	Basic search	In physicians: title/abstract (“medical staff” or “healthcare professionals” or “emergency medical technicians” or physicians) and title/abstract (covid-19 or “covid-19 pandemic” or “sars-cov-2 infection” or “coronavirus disease”) and title/abstract (“sleep wake disorder” OR “sleep disorder” or sleep OR “Sleep Initiation and Maintenance Disorders” OR insomnia OR DIMS OR “sleep quality” OR “sleep hygiene”)	26.06.2020	37
		In nurses: title/abstract (health personal or “healthcare workers” or “emergency medical technicians” or nurse) and title/abstract (covid-19 or “covid-19 pandemic” or “sars-cov-2 infection” or “coronavirus disease”) and title/abstract (“sleep wake disorder” OR “sleep disorder” or sleep OR “Sleep Initiation and Maintenance Disorders” OR insomnia OR DIMS OR “sleep quality” OR “sleep hygiene”)		
CINHAL with EBSCOHOST	Basic search	In physicians: (health personnel “or “medical staff” or “emergency medical technicians” or physicians) and (covid-19 or “covid-19 pandemic” or “sars-cov-2 infection” or “coronavirus disease”) and (“sleep wake disorder” OR “sleep disorder” or sleep OR “Sleep Initiation and Maintenance Disorders” OR insomnia OR “sleep quality” OR “sleep hygiene”)	26.06.2020	9
		In nurses: (“healthcare workers” or “medical staff” or “healthcare professionals” or “emergency medical technicians” or nurse) and (covid-19 or “covid-19 pandemic” or “sars-cov-2 infection” or “coronavirus disease”) and (“sleep wake disorder” OR “sleep disorder” or sleep OR “Sleep Initiation and Maintenance Disorders” OR insomnia OR “sleep quality” OR “sleep hygiene”)		
Medline with EBSCOHOST	Basic search	In physicians: (“healthcare workers” or “medical staff” or physicians) and (covid-19 or “covid-19 pandemic” or “sars-cov-2 infection” or “coronavirus disease”) and (“sleep wake disorder” OR “sleep disorder” or sleep OR “Sleep Initiation and Maintenance Disorders” OR insomnia OR “sleep quality” OR “sleep hygiene”)	26.06.2020	56
		In nurses: (health personnel “or “healthcare professionals” or “emergency medical technicians” or nurse) and (covid-19 or “covid-19 pandemic” or “sars-cov-2 infection” or “coronavirus disease”) and (“sleep wake disorder” OR “sleep disorder” or sleep OR “Sleep Initiation and Maintenance Disorders” OR insomnia OR “sleep quality” OR “sleep hygiene”)		
Science direct	Advanced search	In physicians: (“health personnel” OR “healthcare workers” OR physicians) AND (“sars-cov-2 infection” OR “coronavirus disease” OR covid19) AND (“sleep disorder” OR sleep or insomnia)	24.06.2020	43
		In nurses: (“health personnel” OR “healthcare workers” OR nurse) AND (“sars-cov-2 infection” OR “coronavirus disease” OR covid19) AND (“sleep disorder” OR sleep or insomnia)		
Google scholar	Basic search, search in 2020 regardless of citation and patents	In physicians: (“health personnel” OR “healthcare workers” OR physicians) AND (“sars-cov-2 infection” OR “coronavirus disease” OR covid19) AND (“sleep disorder” OR sleep or insomnia)	24.06.2020	408
		In nurses: (“health personnel” OR “healthcare workers” OR nurse) AND (“sars-cov-2 infection” OR “coronavirus disease” OR covid19) AND (“sleep disorder” OR sleep or insomnia)		

### Study selection procedure

After completing the search process as indicated above, in order to maximize the comprehensiveness of the search, the grey literature and the lists of references in the identified articles were manually reviewed. Initially, the duplicate papers that were identified within various databases were excluded. Subsequently, a list of remaining titles was prepared, for further evaluation. In the first stage, i.e. screening, the title and abstract of the remaining articles were carefully examined and irrelevant studies were excluded, by considering the exclusion criteria. In the second stage, i.e. eligibility evaluation, the full texts of the remaining articles were examined based on the inclusion and exclusion criteria, and similarly, a number of unrelated studies were omitted. To prevent bias, all stages of resource review and data extraction were performed by two reviewers independently. If an article was not included, the reason for the exclusion was mentioned. In cases where there was a disagreement between two reviewers, the third person reviewed the article.

### Quality evaluation

In order to evaluate the quality of articles (i.e. methodological validity and results), a checklist appropriate to the type of study was used. STROBE checklists are commonly used to critique and evaluate the quality of observational studies. The STROBE checklist consists of six scales/general sections that are: title, abstract, introduction, methods, results, and discussion. Some of these scales have subscales, resulting in a total of 32 subscales/items. Each of the 32 items represent different methodological aspects of the study; the subscales include: title, problem statement, objectives, study type, statistical community, sampling strategy, sample size determination, definition of variables and procedures, data collection methods, statistical analysis methods, and findings. Accordingly, the maximum score that could be obtained using the STROBE 32 checklist is 32. Considering the score of 16 as the cut-off point, articles with scores of 16 or above were considered as medium or high-quality articles. In the present study, based on the evaluation conducted using the STROBE checklist, 7 articles entered the systematic review and meta-analysis process (45).

### Statistical analysis

The I^2^ test was used to evaluate the heterogeneity of the selected studies. In order to investigate the publication bias, due to the high volume of samples entered into the study, the Egger’s test and corresponding Funnel plots were adopted with a significance level of 0.05. Data analysis was performed using the Comprehensive Meta-Analysis (version 2) software.

## Results

**Fig. 2:**
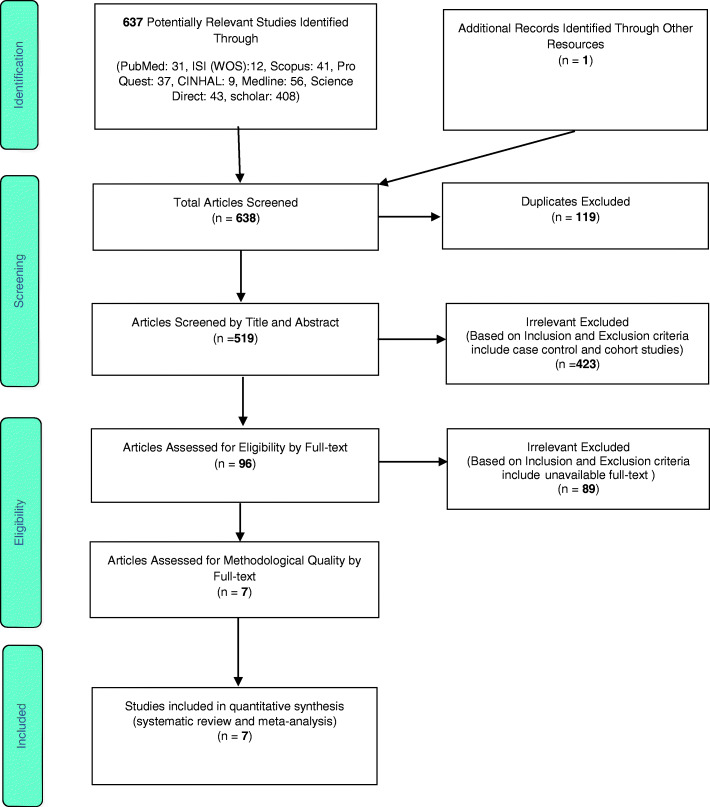
PRISMA flow diagram

Following a systematic search of research repositories and databases, a total of 637 articles were identified and added to the EndNote bibliography management software. After excluding 119 duplicate articles, the title and abstract of 519 articles were reviewed according to the inclusion and exclusion criteria, and after excluding further irrelevant studies, 96 articles were kept for further review and evaluation. At this stage, the full text of the articles was reviewed in accordance with the inclusion and exclusion criteria, and using the STROBE checklist, 7 articles were selected as medium or high-quality articles (Fig. 2).

STROBE checklist was used to review studies. This checklist contains 22 sections, 18 of which are general and practical for all observational studies, including cohort, case study, and cross-sectional ones. Four sections are specific, depending on the type of study, and various aspects of the methodology including objectives of the study, determining the appropriate sample size, type of study, sampling method, research population, data collection method, variables definition and sample study method, data collection tools, objectives of the study, statistical test, and study results.

The information obtained from these studies were used to approximate the prevalence of sleep disturbances in nurses and physicians in hospitals exposed to COVID-19.

### Characteristics of the collected studies

Table 2 provides the general information and characteristics of the selected articles. All of the studies used were cross-sectional. Data had been collected online in 6 studies using a smartphone or via Google Forms. In one study, hospital-based survey data had been collected [25]. Six studies were conducted in China and only one in Iraq. The percentage of women in the study varied from 12 to 100%, and all of the participants were over 18 years old. All collected pieces of research had used self-reporting questionnaires. The Sleep disturbances Severity Index (ISI) questionnaire was used in 3 studies [16, 25, 26], the other 3 studies used the Pittsburgh Sleep Quality Index (PSQI) questionnaire [27–29] and the Athens Sleep disturbances Scale (AIS) questionnaire was used in one study [24]. Further characteristics and information related to these studies are provided in Table 2. A total of 3745 nurses and 2123 physicians were evaluated in all studies, the information of which are presented in Table 3.

**Table 2:** Summary of characteristics of included studies (WeChat: WeChat is the most popular social media platform in China, SD: standard deviation, AIS: Athens Sleep disturbances Scale, ISI: Sleep disturbances Severity Index, PSQI: Pittsburgh Sleep Quality Index

Rows	Name and year	Region	Type of study and data collection method	Workplace samples	Total participants	Mean age(SD) or range of age	Female%	Nurses participants	Physicians participants	Assessment	Cutoff point	Prevalence of insomnia%	
												Nurses	Physicians
1	Abdulah, D. M.2020 [24]	Iraq	Cross-sectional web based google form	pediatric, emergency, special corona, and maternity and gynecology hospitals in Dehuk	268	35.06(7.61)	29.1	_	268	AIS	> 6	_	68.3
2	Lai, J. B.2020 [25].	China	Cross- sectional hospital-based survey	Hospitals in Wuhan, Hubei province outside Wuhan, and Outside Hubei province	1257	(26-40)	76.6	764	493	ISI	> 8	38.2	27.4
3	Que, J. Y. 2020 [26].	China	Cross -sectional social media platform- based WeChat	Healthcare workers from different regions throughout China	2285	31.06(6.99)	69.06	208	860	ISI	> 8	33.2	30.9
4	Wang, S. 2020 [27].	China	Cross –sectional media platform-based smartphone	Children’s Healthcare Centre of Renmin Hospital of Wuhan	123	33.75(8.41)	90	75	48	PSQI	> 7	24	60.4
5	Zhang, C. X.2020 [16].	China	Cross sectional questionnaire delivered through the mobile phone, WeChat	hospital staff from all over China, including the frontline medical workers in Wuhan	1563	> 18	82.7	984	454	ISI	> 8	40.1	27.3
6	Tu, Zhi-hao.2020 [28]	China	Cross sectional questionnaire delivered through the mobile phone, WeChat	“Huoshenshan” Hospital in Wuhan	100	34.44(5.85)	100	100	_	PSQI	> 7	60	_
7	Zhou, Y.2020 [29].	China	Cross –sectional using the Wenjuanxing program which is an application embedded with WeChat,	hospital in Liaoning province, China	1931	35.08(8.04)	12	1614	_	PSQI	> 7	19.5	_

**Table 3:** Meta-analysis of the prevalence of sleep disturbances among hospital nurses and physicians during the COVID-19 outbreak

Job	Total of participants	Prevalence%	Confidence interval 95%	I^2^	Significant bias (Egger’s test)
Nurses	3745	34.8	(24.8-46.4)	97.4	P = 0.618
Physicians	2123	41.6	(27.7-57)	97.3	P = 0.322

### Evaluating heterogeneity and publication bias

**Fig. 3:**
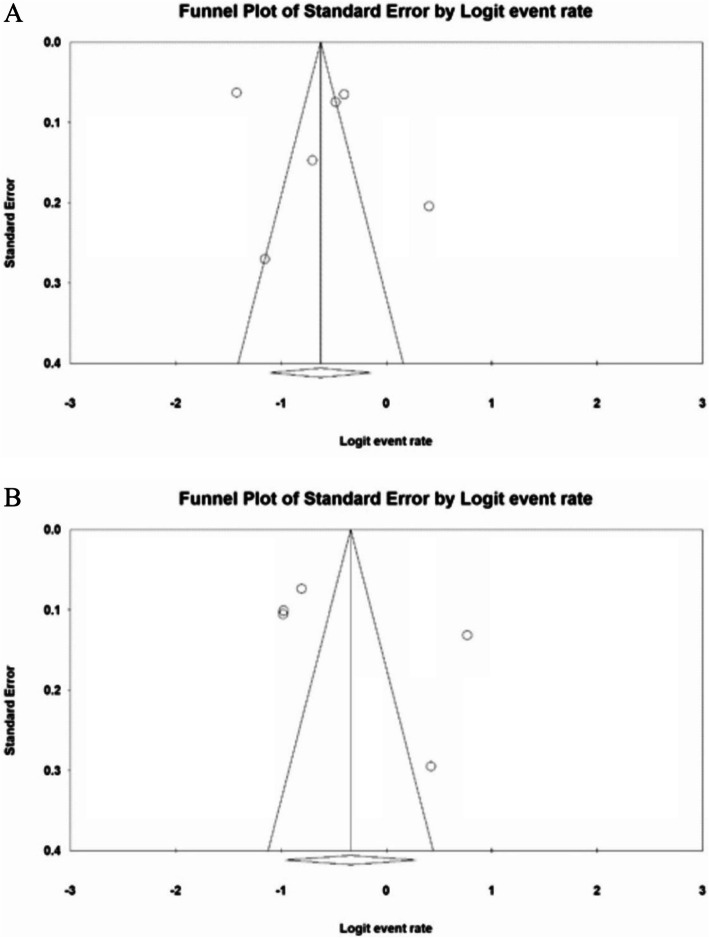
**a** Funnel plot of the reported results of sleep disorder among hospital nurses faced with the COVID-19 patients, **b** Funnel plot of the reported results of sleep disorder among hospital physicians faced with the COVID-19 patients

Based on the results of I^2^ test, the prevalence of sleep disturbances in nurses and physicians is I^2^: 97.4% and I^2^: 97.3% respectively, and due to the heterogeneity of the selected studies, the random effects model was used to combine the reported results of studies and approximate the total prevalence. To assess the publication bias, funnel diagram and Egger’s test at a significance level of 0.05 were used that showed no bias in this study (*P* = 0.618, *P* = 0.322) (Fig. 3).

### Prevalence of sleep disturbances

**Fig. 4:**
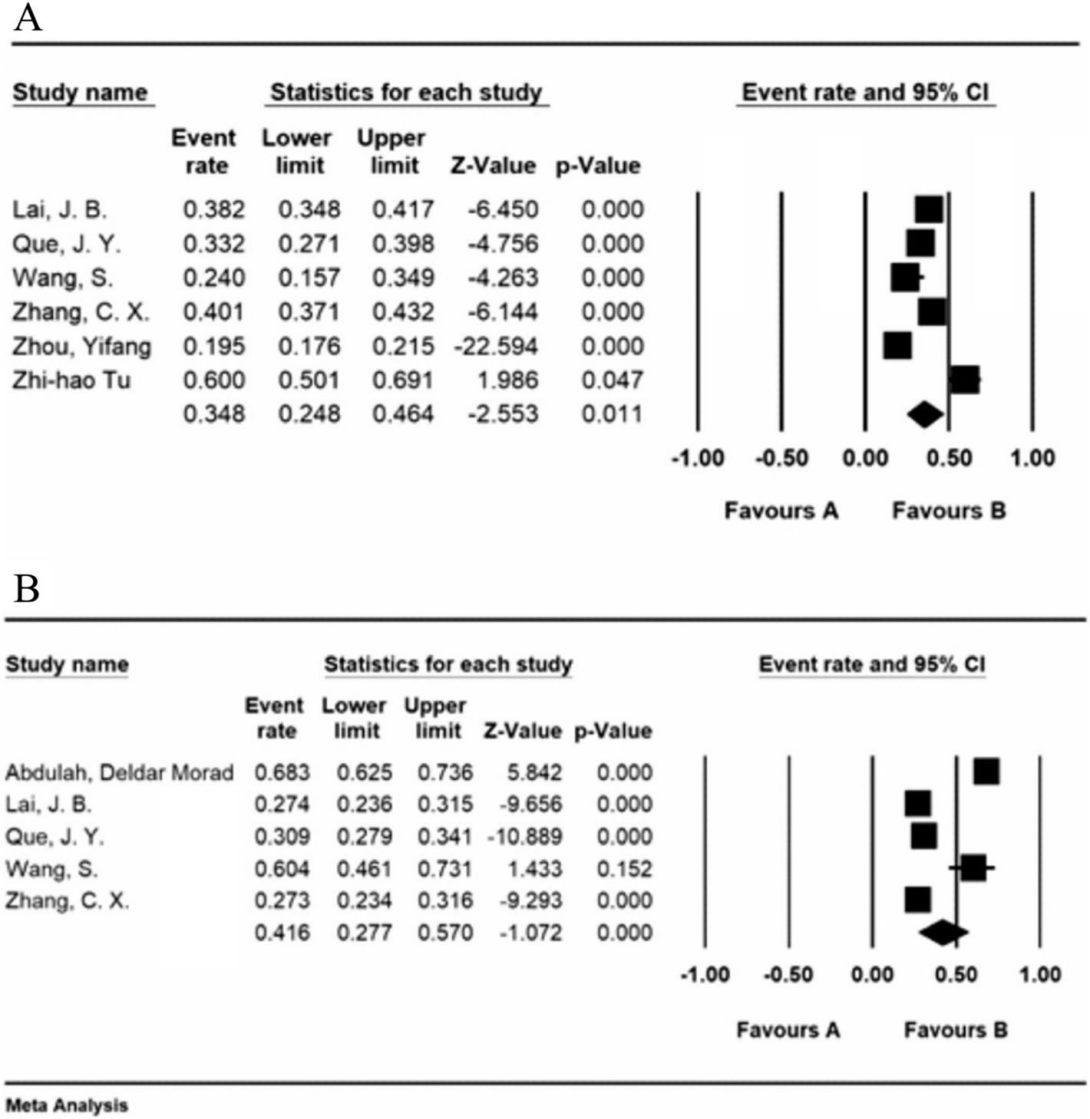
**a** The prevalence of sleep disturbances among hospital nurses facing the COVID-19 patients (95% confidence interval), **b** The prevalence of sleep disturbances among hospital physicians facing the COVID-19 patients (95% confidence interval)

According to our study, the prevalence of sleep disturbances among nurses is 34.8% (95% CI: 24.8-46.4%). The lowest prevalence reported in the research conducted by Zhou et al. (2020) and was reported as 19.5% (95% CI: 17.6-21.54%) [29]. Tu et al. (2020) reported the highest prevalence of sleep disturbances among nurses with 60% (95% CI: 50.1-69.1%) [28]. The prevalence of sleep disturbances in physicians was reported 41.6% (95% CI: 27.7-57%). Considering the physicians’ group, the lowest prevalence of sleep disturbances as reported in Zhang et al. (2020) was 27.3% (95% CI: 23.4-31.6%) [16]. The highest prevalence was also observed in the study of Abdulah & Musa (2020) with 68.3% (95% CI: 62.5-73.6%) [24]. Figure 4 illustrates the results of the performed meta-analysis. The center point of each line denotes the prevalence in each study, and the diamond shape presents the total prevalence by considering the results of all studies combined (Fig. 4).

### Sensitivity analysis

**Fig. 5:**
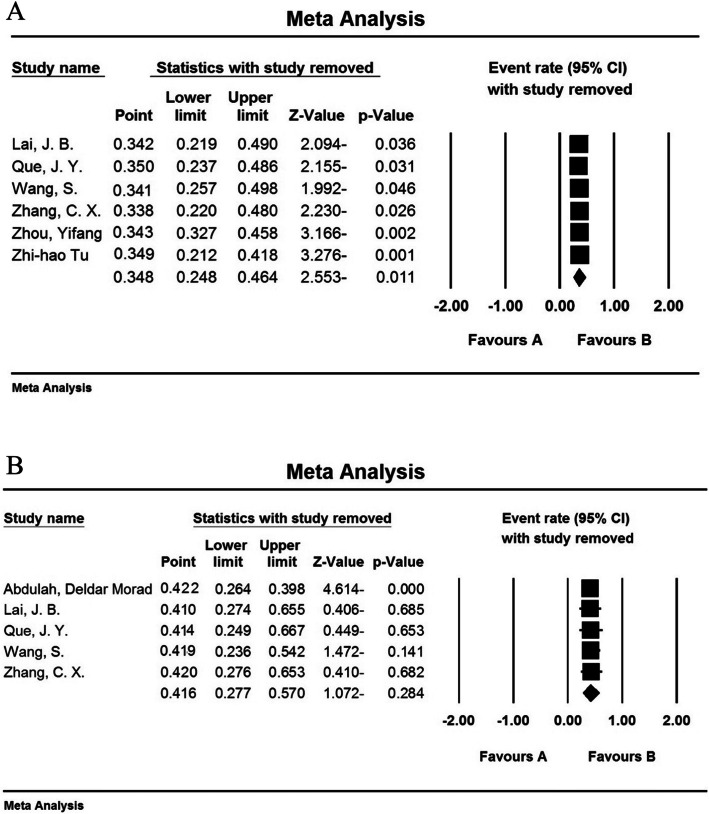
Results of sensitivity analysis among hospital nurses (5A) and physicians (5B) exposed to the COVID-19

A sensitivity analysis was perfumed to ensure the stability results, after removing each study results did not change in sleep disturbances among nurses (Fig. 5a) and did not change in sleep disturbances among physicians (Fig. 5b).

### Meta-regression test

**Fig. 6:**
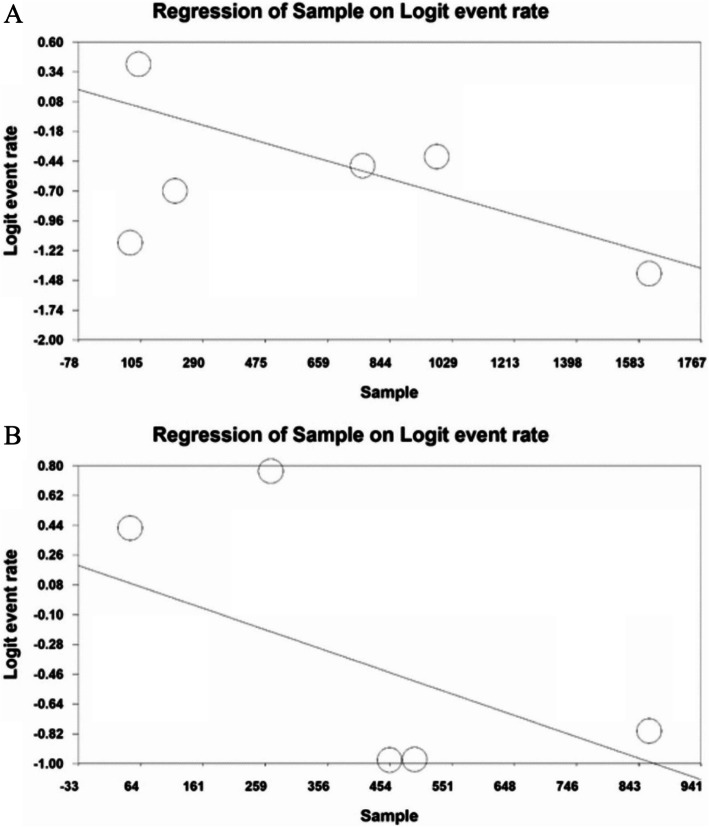
**a** The meta-regression chart of sleep disturbances prevalence among hospital nurses exposed to the COVID-19 patients by sample size, **b** The meta-regression chart of sleep disturbances prevalence among hospital physicians exposed to the COVID-19 patients by sample size

**Fig. 7:**
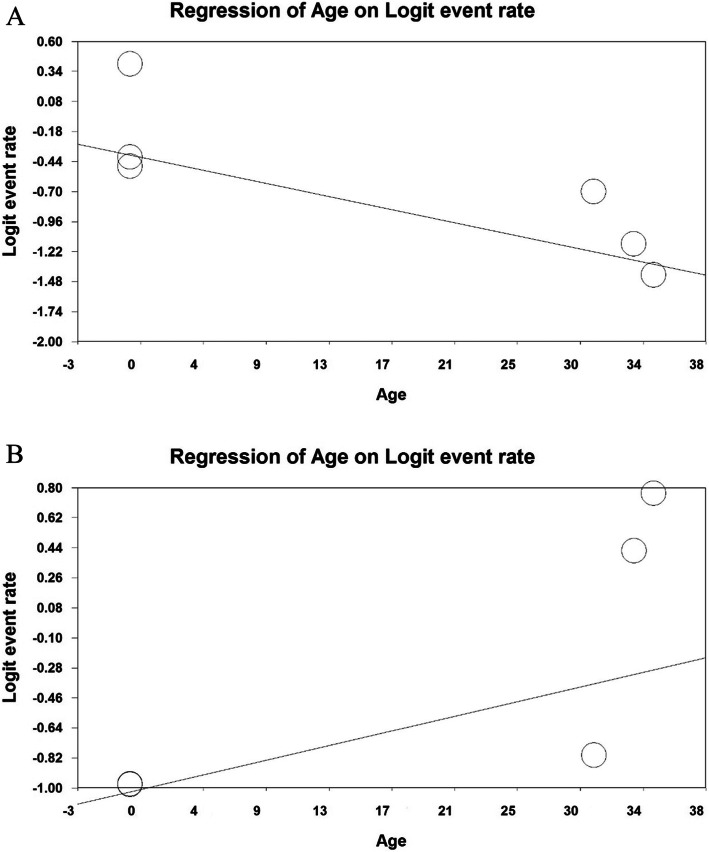
**a** The meta-regression chart of sleep disturbances prevalence among hospital nurses exposed to the COVID-19 patients by age of nurses, **b** The meta-regression chart of sleep disturbances prevalence among hospital physicians exposed to the COVID-19 patients by age of physicians

In order to investigate the impacts of variables affecting the heterogeneity of sleep disturbances prevalence among physicians and nurses, meta-regression was used to assess the study effect size (Figs. 6 and 7). According to Fig. 6a, the prevalence of sleep disturbances among nurses decreases with increasing the sample size, and this is statistically significant (*P* < 0.05). Moreover, considering Fig. 6b, sleep disturbances among physicians decreases with increasing the sample size, which is statistically significant (*P* < 0.05).

According to Fig. 7a, the prevalence of sleep disturbances among nurses decreases with increasing the age of nurses, and this is statistically significant (*P* < 0.05). Moreover, considering Fig. 7b, sleep disturbances among physicians increases with increasing the age of physicians, which is statistically significant (*P* < 0.05).

## Discussion

To date, several studies have examined the effects of exposure to COVID-19 patients on sleep disturbances in nurses and physicians. However, these studies lack an overarching and systematic evaluation and critique. Therefore, the present systematic review and meta-analysis was conducted for the first time with the aim of determining the prevalence of sleep disturbances in hospital nurses and physicians facing the COVID-19 patients. Following the PRISMA guidelines and considering the inclusion and exclusion criteria, 7 cross-sectional studies were selected for analysis. The meta-analysis findings demonstrate that the prevalence of sleep disturbances among the nurses and physicians facing the COVID-19 patients is 34.8 and 41.6% respectively.

The results of the analysis also showed that the prevalence of sleep disturbances in physicians is higher than nurses. Nevertheless, this was not consistent with most of the existing studies [16, 25, 26]. On the other hand, the results of the study by Wang et al. (2020) [27] were consistent with our finding. Since the population of the physicians studied in this meta-analysis was lower than that of the nurses, the effect of this difference on the prevalence of sleep disturbances in physicians could not be ignored.

A healthy sleep has an important effect on human performance. Sleep disorder can cause drowsiness, fatigue and loss of appetite. On the other hand, this disorder reduces concentration and can cause workplace errors and accidents [30]. Today, nurses and physicians are at the forefront of the fight against COVID-19. Moreover, these groups are always exposed to factors such as long-term shifts, high risk of infection, and, in some cases, lack of medical equipment. These challenges increase anxiety and psychological trauma. Previous studies have shown that exposure to work shifts and unusual work schedules causes sleep disturbances, and the prevalence of sleep disturbances in night shift nurses has been reported as 32.6% [31, 32].

Epidemics have always been a factor impacting the mental health of health care workers. After the outbreak of severe acute respiratory syndrome (SARS), more than 8000 people around the world contracted the disease from 2002 to2003, of which 1707 were medical staff members [33]. A study by McAlonan et al. (2007) showed that healthcare staff facing the SARS patients had higher stress scores than employees who did not face the patients. The group also suffered from fatigue, anxiety, and sleep disturbances [34]. The prevalence of sleep disturbances among the affected healthcare workers in Taiwan during the SARS outbreak was reported to be 28.4% [35]. Pappa et al. (2020) conducted a meta-analysis to examine the prevalence of sleep disturbances among healthcare staff facing the COVID-19 patients; the study reported the prevalence of sleep disturbances among the studied population as 38.8% [36].

Research have shown that the prevalence of sleep disturbances during exposure to COVID-19 has increased than normal times [37]. A study by Abdulah & Musa (2020) reported that the prevalence of sleep disturbances in Iraqi physicians exposed to COVID-19 is 68.3%, while a similar study was conducted a year earlier, in 2019, in the same region and had reported the prevalence of sleep disturbances as 45.5% [24].

In many countries around the world, especially in the developed nations, healthcare workers are exposed to severe job stresses and are at a greater risk off developing mental health disorders than in other occupations. Nurses are known to be the largest group of healthcare workers. Anxiety, depression, substance abuse, aggression and burnout are the most common physical and mental disorders observed in this group [38]. Stress and depression have also been reported in other groups of medical staff. This increases the incidence of other mental disorders, and also intensifies the likelihood of impaired professional behavior [39]. Stress, anxiety, and other mental health problems can reduce the quality of sleep in nurses and physicians [40].

The results of the analysis also showed that the prevalence of sleep disturbances among nurses and physicians decreases with increasing the sample size, this situation indicates an increase in the number of nurses and physicians under study and an increase in the denominator of the prevalence fraction and can reduce the prevalence of the study. Also the prevalence of sleep disturbances among nurses decreases with increasing the age of nurses and sleep disturbances among physicians increases with increasing the age of physicians, Increasing the age of nurses and physicians is associated with an increase in physical problems and fatigue from work, and such a situation increases the need for rest and sleep in older nurses and physicians.

There are several factors that can contribute to sleep disturbances among healthcare workers that are treating the COVID-19 patients. One of these factors is the stress of getting infected. On the other hand, the experience of past epidemics suggests that many medical staff are likely to be quarantined due to the exposure to the disease. The main pressure caused by epidemics was also placed mainly on healthcare workers; for instance, during an epidemic one out of six nurses showed severe symptoms of stress and anxiety. Studies also emphasize that symptoms of stress are more common in people with sleep disturbances than in the control group [41, 42]. All of the studies examined in this systematic review also stressed on the effects of exposure to COVID-19, and related psychological problems, on increasing sleep disturbances.

Medical staff can be considered as a part of crisis management personnel, and in the crisis caused by the COVID-19 spread, they play a major role in screening and treating the patients. Yet, they will not be immune to the psychological effects of this disease. Failure to provide the necessary care equipment will cause a feeling of ‘lack of proper support’ among the healthcare workers. One of the main challenges with COVID-19 is its rapid transmission; Moreover, it does not have a definite treatment. These may consequently result in frustration, and powerlessness among medical staff [43–45].

### Limitation

There were limitations in this study; for instance, all of the studies were cross-sectional. This prevented us to examine the sleep disturbances prevalence in different time intervals, or to evaluate all factors that can increase or decrease mental health disorders among the study populations. Apart from one study, others examined sleep disturbances among the Chinese nurses and physicians. Therefore, it may not be possible to generalize the results to nurses and physicians in other regions or countries. On the other hand, a number of studies were visible as pre-prints and were removed from our collection, since their review process had not yet been completed. Research works that did not specify the prevalence of sleep disturbances in healthcare workers staff (separately by the staff occupation/role) were also excluded from this work. Finally, due to the use of self-reporting tools, as well as sampling and completing questionnaires online, it was not possible to review the samples clinically.

## Conclusion

It is clear that the COVID-19 crisis has resulted in several physical and psychological disorders in different societies. The medical staff, as the front line of the fight against COVID-19, are even more vulnerable to the harmful effects of this disease than other jobs and groups in society. Increasing workplace, physical and psychological stresses increases sleep disturbances among the medical staff, especially nurses and doctors, facing the COVID-19 patients. In other words, increased stress and anxiety due to exposure to COVID-19 increases the prevalence of sleep disturbances in nurses and physicians. A future study trajectory would be the examination of the effects of stress and anxiety on sleep disturbances among different healthcare workers.

## Data Availability

Datasets are available through the corresponding author upon reasonable request.

## Abbreviations

SARS: Severe Acute Respiratory Syndrome
MERS: Middle East Respiratory Syndrome
PTSD: Post-Traumatic Stress Disorder
PSQI: Pittsburgh Sleep Quality Index
STROBE: Strengthening the Reporting of Observational studies in Epidemiology
PRISMA: Preferred Reporting Items for Systematic Reviews and Meta-Analysis.

## References

[1] Zheng Y-Y (2020). COVID-19 and the cardiovascular system. Nat Rev Cardiol.

[2] Velavan TP, Meyer CG (2020). The COVID-19 epidemic. Trop Med Int Health.

[3] Fang Y, et al. Sensitivity of Chest CT for COVID-19: Comparison to RT-PCR. Radiology. 2020;296(2):200432. 10.1148/radiol.2020200432 PMC7233365 32073353

[4] Amerio A (2020). Covid-19 pandemic impact on mental health: a web-based cross-sectional survey on a sample of Italian general practitioners. Acta Bio Med.

[5] Lipsitch M, Swerdlow DL, Finelli L (2020). Defining the epidemiology of Covid-19 — studies needed. N Engl J Med.

[6] Jin Y (2020). Virology, epidemiology, pathogenesis, and control of COVID-19. Viruses.

[7] Chen Q (2020). Mental health care for medical staff in China during the COVID-19 outbreak. Lancet Psychiatry.

[8] Rastogi Y, et al. The novel coronavirus 2019-nCoV: its evolution and transmission into humans causing global COVID-19 pandemic. Int J Environ Sci Technol. 2020;26:1–8. 10.1007/s13762-020-02781-2 PMC7247958 32837521

[9] Shereen MA (2020). COVID-19 infection: origin, transmission, and characteristics of human coronaviruses. J Adv Res.

[10] Zhu FC, Li YH, Guan XH, Hou LH, Wang WJ, Li JX, Wu SP, Wang BS, Wang Z, Wang L, Jia SY, Jiang HD, Wang L, Jiang T, Hu Y, Gou JB, Xu SB, Xu JJ, Wang XW, Wang W, Chen W. Safety, tolerability, and immunogenicity of a recombinant adenovirus type-5 vectored COVID-19 vaccine: a dose-escalation, open-label, non-randomised, first-in-human trial. Lancet. 2020;395(10240):1845–54. 10.1016/S0140-6736(20)31208-3 PMC7255193 32450106

[11] Terpos E (2020). Hematological findings and complications of COVID-19. Am J Hematol.

[12] Qiu J (2020). A nationwide survey of psychological distress among Chinese people in the COVID-19 epidemic: implications and policy recommendations. General Psychiatry.

[13] Yifan T, et al. Symptom cluster of ICU nurses treating COVID-19 pneumonia patients in Wuhan, China. J Pain Symptom Manag. 2020;60(1):e48–e53. 10.1016/j.jpainsymman.2020.03.039 PMC7141465 32276095

[14] Blake H, et al. Mitigating the psychological impact of COVID-19 on healthcare workers: a digital learning package. Int J Environ Res Public Health. 2020;17(9):2997–3003. 10.3390/ijerph17092997 PMC7246821 32357424

[15] Dong Z-Q, et al. The social psychological impact of the COVID-19 epidemic on medical staff in China: a cross-sectional study. Eur Psychiatry. 2020;63(1):1–22. 10.1192/j.eurpsy.2020.59 PMC7343668 32476633

[16] Zhang CX, et al. Survey of sleep disturbances and related social psychological factors among medical staff involved in the 2019 novel coronavirus disease outbreak. Front Psychiatry. 2020;11:306-12. 10.3389/fpsyt.2020.00306 PMC7171048 32346373

[17] Geoffroy PA (2020). Psychological support system for hospital workers during the Covid-19 outbreak: rapid design and implementation of the Covid-Psy hotline. Front Psychiatry.

[18] Wu KL, Wei XM. Analysis of psychological and sleep status and exercise rehabilitation of front-line clinical staff in the fight against COVID-19 in China. Med Sci Monit Basic Res. 2020;26:e924085. 10.12659/MSMBR.924085 PMC7241216 32389999

[19] Huang YE, Zhao N. Generalized anxiety disorder, depressive symptoms and sleep quality during COVID-19 outbreak in China: a web-based cross-sectional survey. Psychiatry Res. 2020;288:112954–63. 10.1016/j.psychres.2020.112954 PMC7152913 32325383

[20] Léger D, Bayon V (2010). Societal costs of sleep disturbances. Sleep Med Rev.

[21] Kousloglou S (2014). Sleep disturbances and burnout in Greek nurses. Hippokratia.

[22] Silva-Costa A, Griep RH, Rotenberg L (2015). Associations of a short sleep duration, insufficient sleep, and sleep disturbances with self-rated health among nurses. PLoS One.

[23] Yazdi Z (2014). Sleep quality and sleep disturbances in nurses with different circadian chronotypes: Morningness and eveningness orientation. Work.

[24] Abdulah DM, Musa DH. Sleep disturbances and Stress of Physicians during COVID-19 Outbreak. Sleep Med X. 2020:100017. 10.1016/j.sleepx.2020.100017 PMC7237900 33860222

[25] Lai JB, et al. Factors associated with mental health outcomes among health care workers exposed to coronavirus disease 2019. JAMA Network Open. 2020;3(3):e203976. 10.1001/jamanetworkopen.2020.3976 PMC7090843 32202646

[26] Que JY, et al. Psychological impact of the COVID-19 pandemic on healthcare workers: a cross-sectional study in China. Gene Psychiatry. 2020;33(3)::e100259. 10.1136/gpsych-2020-100259 PMC7299004 32596640

[27] Wang, S., et al., Sleep disturbances among medical workers during the outbreak of COVID-2019. Occupational medicine (Oxford, England), 2020;kqaa074. 10.1093/occmed/kqaa074 PMC7239094 32372077

[28] Tu Z-h, He J-w, Zhou N. Sleep quality and mood symptoms in conscripted frontline nurse in Wuhan, China during COVID-19 outbreak: a cross-sectional study. Medicine. 2020;99(26):e20769. 10.1097/MD.0000000000020769 PMC7328950 32590755

[29] Zhou Y (2020). Prevalence and demographic correlates of poor sleep quality among frontline health professionals in Liaoning Province, China during the COVID-19 outbreak. Front Psychiatry.

[30] Zdanowicz T (2020). Sleep disturbances, sleepiness, and fatigue among polish nurses. Workplace Health Saf.

[31] Zhang W (2020). Mental health and psychosocial problems of medical health workers during the COVID-19 epidemic in China. Psychotherapy and psychosomatics.

[32] Tsai K, Lee T-Y, Chung M-H (2017). Sleep disturbances in female nurses: a nationwide retrospective study. Int J Occup Saf Ergon.

[33] Chan-Yeung M (2004). Severe acute respiratory syndrome (SARS) and healthcare workers. Int J Occup Environ Health.

[34] McAlonan GM (2007). Immediate and sustained psychological impact of an emerging infectious disease outbreak on health care workers. Can J Psychiatry.

[35] Su T-P (2007). Prevalence of psychiatric morbidity and psychological adaptation of the nurses in a structured SARS caring unit during outbreak: a prospective and periodic assessment study in Taiwan. J Psychiatr Res.

[36] Pappa S, et al. Prevalence of depression, anxiety, and sleep disturbances among healthcare workers during the COVID-19 pandemic: a systematic review and meta-analysis. Brain Behav Immun. 2020;88:901–7. 10.1016/j.bbi.2020.05.026 PMC7206431 32437915

[37] Li Y (2020). Sleep disturbances and psychological reactions during the COVID-19 outbreak in China. J Clin Sleep Med.

[38] Perry L (2015). The mental health of nurses in acute teaching hospital settings: a cross-sectional survey. BMC Nurs.

[39] Compton MT, Frank E (2011). Mental health concerns among Canadian physicians: results from the 2007-2008 Canadian physician health study. Compr Psychiatry.

[40] Patterson PD (2012). Association between poor sleep, fatigue, and safety outcomes in emergency medical services providers. Prehospital Emergency Care.

[41] Zhang C (2020). Survey of sleep disturbances and related social psychological factors among medical staff involved in the 2019 novel coronavirus disease outbreak. Front Psychiatry.

[42] Wu KK, Chan KS. The development of the Chinese version of Impact of Event Scale–Revised (CIES-R). Soc Psychiatry Psychiatr Epidemiol. 2003;38(2):94–8. 10.1007/s00127-003-0611-x. 10.1007/s00127-003-0611-x 12563552

[43] Spoorthy MS, Pratapa SK, Mahant S (2020). Mental health problems faced by healthcare workers due to the COVID-19 pandemic-a review. Asian J Psychiatr.

[44] Moll SE (2014). The web of silence: a qualitative case study of early intervention and support for healthcare workers with mental ill-health. BMC Public Health.

[45] Salari N, Mohammadi M, Vaisi-Raygani A, Abdi A, Shohaimi S, Khaledipaveh B (2020). The prevalence of severe depression in Iranian older adult: a meta-analysis and meta-regression. BMC Geriatr.

